# Machine Learning Driven Analysis Yields Insight into the Processing Robustness of Organic Photovoltaics

**DOI:** 10.1021/acsmacrolett.6c00284

**Published:** 2026-07-23

**Authors:** Stephen Wong, Ankush Kumar Mishra, Baskar Ganapathysubramanian, Enrique D. Gomez

**Affiliations:** † Department of Chemical Engineering, The Pennsylvania State University, University Park, Pennsylvania 16802, United States; ‡ Department of Mechanical Engineering, 1177 Iowa State University, Ames, Iowa 50011, United States; § Translational AI Research Center (TrAC), 1177 Iowa State University, Ames, Iowa 50011, United States; ⊥ Materials Research Institute, The Pennsylvania State University, University Park, Pennsylvania 16802, United States

## Abstract

Optimizing the processing window for organic photovoltaics (OPVs) can deliver high efficiency and yield. In practice, small drifts in solution concentration, additive fraction, drying rate, or ambient humidity readily perturb the evolving morphology, making champion devices hard to reproduce. We combine a complementary Taguchi and space-filling design-of-experiments strategy with machine-learning surrogates to map processing robustness in PM6:Y6 films using acetone and 1-chloronaphthalene as the processing additives. Robustness is quantified via parameter-space fraction (share of conditions exceeding a power conversion efficiency threshold), persistence curves (how that share contracts as the threshold tightens), and fill fraction (compactness of high-performing regions). The parameter space landscape when using acetone as a solvent additive exhibits a broad, robust regime, with around 73% of the explored space exceeding 9% PCE; this is consistent with our hypothesis that faster evaporation yields morphology tolerant to routine variability. This ML-enabled framework disentangles manufacturing-relevant robustness from peak PCE and offers a general, data-efficient way to compare processing-window robustness with reduced experimental effort. Such tools integrate naturally with self-driving laboratories and can identify new processing opportunities for OPVs.

## Graphical abstract

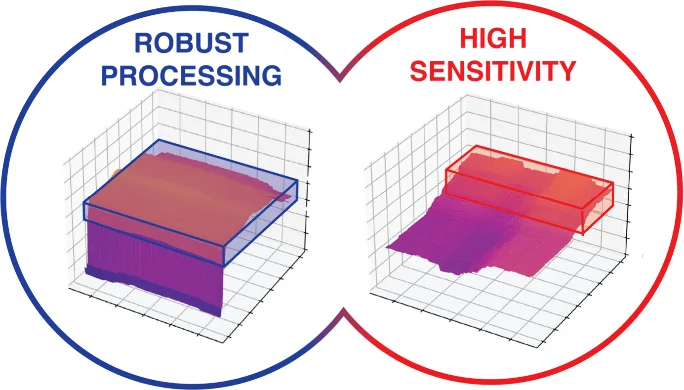

Substantial work has gone into the development of new organic photovoltaic materials, understanding active layer microstructure, and identifying the mechanisms behind how chemistry and structure relate to electronic properties.^1–15^ Properties including Hansen solubility parameters,^16–18^ relative boiling points,^19^ and solvent volatility^20,21^ each influence film formation, and by extension the efficiency of resulting photovoltaic devices. Yet the multivariate, coupled nature of chemistry and processing renders *a priori* prediction of power conversion efficiency (PCE) challenging.^22,23^

Consequently, maximizing device performance requires exploring many variables and fine-tuning multiple processing conditions, yielding a high-dimensional parameter space. For any material combination, one must delineate regions that meet target performance from those that do not. Evaluating new materials or processing perturbations, including the introduction of additives, demands substantial experimental time and resources to determine their true effect on performance.

For scaling beyond lab-scale champion devices, reproducibility and yield hinge on identifying processing windows that remain high-performing under routine variability. Modest drifts in solution concentration, additive fraction, drying rate, or ambient humidity can disrupt intended morphology evolution, shrinking the set of conditions that reliably achieve a target PCE. We therefore focus on processing robustness, i.e., asking how much of the high-dimensional space maintains device quality under such perturbations. We quantify robustness with three complementary metrics: (i) parameter-space fraction (share of conditions exceeding a PCE threshold), (ii) persistence curves (how that share contracts as the threshold tightens), and (iii) fill fraction (the compactness/contiguity of high-performing regions). Framed this way, our robustness analysis supports the prediction of manufacturing yield and tolerance requirements. Thus, a framework that combines broad experimental sampling with interpretable modeling is needed to capture both local optima and global robustness.

Design of experiments (DOE), originating in agriculture and widely used in chemical engineering, has long enabled efficient screening and optimization.^24,25^ In OPVs, response surface methods and DOE have been deployed to accelerate recipe optimization.^26–28^ Studies that combine DOE with machine learning (ML) largely emphasize locating (local) optima.^29–31^ These strategies are effective for maximizing peak PCE but provide only a localized view of the processing landscape and offer limited guidance on how broad or compact the truly viable region is. In parallel, ML efforts that screen materials via molecular descriptors^22,23,32–34^ can underemphasize the influence of processing parameters on power conversion efficiency.

In this work, we pair DOE with ML surrogates explicitly as a computational aid for exploration and mapping the OPV processing space and for quantifying robustness with the three metrics stated above. Using poly­[(2,6-(4,8-bis­(5-(2-ethylhexyl)-4-fluorothiophen-2-yl)-benzo­[1,2-b:4,5-b’]­dithiophene))-alt-(5,5-(1’,3’-di-2-thienyl-5’,7’-bis­(2-ethylhexyl)­benzo­[1’,2’-c:4’,5’-c’]­dithiophene-4,8-dione))]­(PM6) and 2,2'-((2Z,2'Z)-((12,13-bis­(2-ethylhexyl)-3,9-diundecyl-12,13-dihydro-[1,2,5]­thiadiazolo­[3,4-e]­thieno­[2",3’':4’,5']­thieno­[2',3':4,5]­pyrrolo­[3,2-g]­thieno­[2',3':4,5]­thieno­[3,2-b]­indole-2,10-diyl)­bis­(methanylylidene))­bis­(5,6-difluoro-3-oxo-2,3-dihydro-1H-indene-2,1-diylidene))­dimalononitrile­(Y6) with acetone or 1-chloronapthalene as a processing additive, we show that the parameter space when acetone is used as an additive contains a broad, contiguous robust regime, while also illustrating how the framework distinguishes manufacturing-relevant robustness from record PCE. The approach is general and data-efficient, requiring no additional physical experimentation beyond the planned DOE, and integrates naturally with self-driving laboratories to inform closed-loop recipe development.

We used PM6:Y6 as a model system due to their high performance when used in OPV active layers.^11,35–39^ We use acetone and 1-chloronaphthalene as solvent additives for processing of PM6:Y6 films to compare their resulting impacts on photovoltaic device processing, and to examine the viability of robust processing analysis on multiple systems. The positive impacts of 1-chloronaphthalene on film crystallinity and morphology are well documented.^39–41^ High boiling point additives, however, have also been shown to remain in active layer films, having negative long-term impacts on active layer stability.^42,43^

Fifty-seven different PM6:Y6 processing permutations were tested with acetone as an additive, and 64 points were tested using 1-chloronaphthalene as an additive. Experiments were based on Taguchi design and pseudorandom Sobol sampling for additional coverage (Figure S1; see Supporting Information for additional sampling and design of experiments details). Seven extra points were taken in the 1-chloronaphthalene parameter space to improve model resolution at extreme processing conditions. Each individual permutation was sampled in 2–3 trials, with each trial composed of approximately six devices, resulting in 12–18 total data points per condition. The collective data set has been separated by additive, normalized, and plotted as a histogram in Figure 1, depicting the overall PCE distribution for each additive. The data sets reveal skewed distribution for 1-chloronaphthalene suggesting a higher degree of variance in the resulting devices. 1-chloronaphthalene yielded a higher champion efficiency than acetone, although not as high as literature reports likely due to material batch limitations. Acetone shows a normal distribution with a higher 9.1% mean PCE compared to 6.7% for 1-chloronaphthalene. This trend continues for the third quartile comparison (10.2% and 9.9%, respectively) but reverses near the overall maxima. Most devices using acetone additive outperformed those with 1-chloronaphthalene, with the top quarter of devices continuing that trend despite superior champion performance from OPVs containing 1-chloronaphthalene. Figure 1 highlights differences between the overall distributions but cannot resolve where in the parameter space these outcomes occur, or whether they belong to broad regions of high performance, or multiple isolated peaks. Bridging this interpretability gap requires tools capable of reconstructing the overall geometry of the PCE landscape.

**Figure 1:**
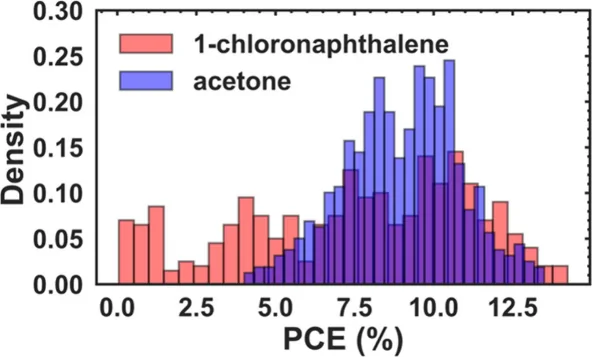
Histogram of training data for PM6:Y6 OPVs with acetone (57 points) and 1-chloronaphthalene (64 points).

Random forest (RF) regression demonstrated the best predictive performance when modeling PM6:Y6 photovoltaics using both 1-chloronaphthalene and acetone (Figure S2 of the Supporting Information). RF is an ensemble machine learning algorithm that averages the output of many parallel decision trees when making predictions. Careful tuning of the tree number and depth can leverage these weak learners into an algorithm more robust to noise and capable of fitting high dimensional training data.^44,45^ Trained models achieved an *R* ^2^ of 0.69 for acetone and *R* ^2^ of 0.31 for 1-chloronaphthalene when evaluated against an 80/20 stratified train test split. These *R* ^2^ values reflect the ability of the model to primarily capture trends within the training data alongside capacity for interpolation across the evaluated parameter space. The low *R* ^2^ for PM6:Y6 devices made with the 1-chloronaphthalene additive is likely due to the wide PCE range and skewed distribution. While the 1-chloronaphthalene model is limited, the surface has been retained as a case study to compare trends in the OPV parameter space, although conclusions regarding the impact of each additive should be confirmed with a higher-quality model for 1-chloronapthalene devices.

PCE landscapes yielded singular champion optima in each parameter space, shown in Figure 2 alongside initial analysis of the nearby parameter space. 1-Chloronaphthalene achieves a predicted maximum PCE of 12.4% (Figure 2a), while acetone reaches 11.8% (Figure 2b). The portion of the PCE landscape visible in Figure 2a,b does not represent the true extent of each local optima due to projecting a high dimension surface into 2D. Connected component analysis was critical for analyzing the size and shape of local optima without reducing dimensionality. The parameter space fraction is the portion of the tested parameter space that produces photovoltaic devices above a given efficiency. This has been plotted alongside the local maximum PCE in Figure 2c. When acetone is used as an additive, 773% of the overall parameter space contains devices with PCE over 9.0% within a single continuous local maximum. The 1-chloronaphthalene local maximum is far smaller, occupying less than one-fifth the parameter space. Comparative analysis of the parameter space fractions, alongside the training data distributions, suggests that the acetone additive yields a larger portion of the parameter space with photovoltaics achieving over 9% PCE.

**Figure 2:**
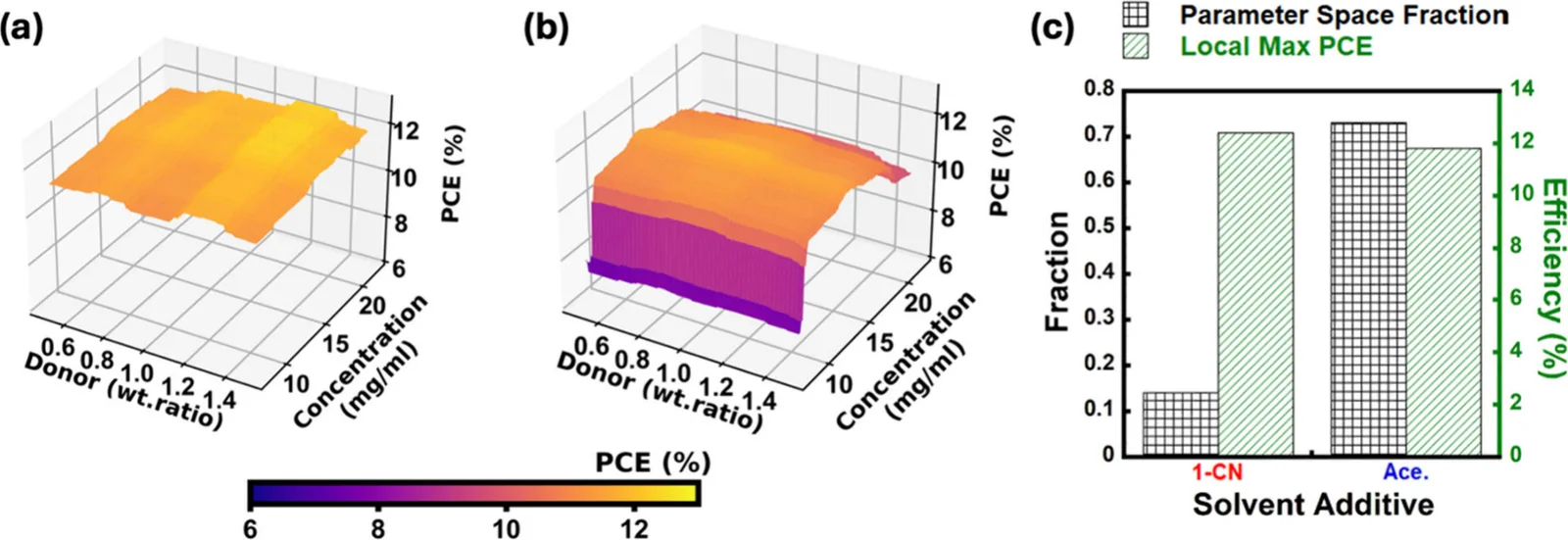
Random forest fits to training data with areas of interest shown: (a) 0.3 v/v% 1-chloronaphthalene, 4400 rpm spin speed and 55 °C annealing temperature; (b) 2.4 v/v% acetone, 3300 rpm, and 87 °C; (c) Parameter space fraction and average power conversion efficiency for devices with PCE > 9.0% using either 1-chloronaphthatalene (1-CN) or acetone (Ace.) as a solvent additive.

By iteratively repeating these analyses at higher threshold efficiencies, we examine how processing robustness persists at processing conditions producing high efficiency. Figure 3a shows that the satisfactory parameter space fraction decreases quickly, reflecting higher requirements for experimental precision as efficiency demands increase. We estimate the error of our prediction by training models on five experimental trials, such that the error bars in Figure 3a are the standard deviation of our predicted values (see Supporting Information for details). By 11% PCE, the acetone parameter space fraction has dropped to about 5%. When targeting OPVs with such high efficiency and low parameter space faction, equipment and protocol precision become key limiting factors for fabrication yield.

**Figure 3:**
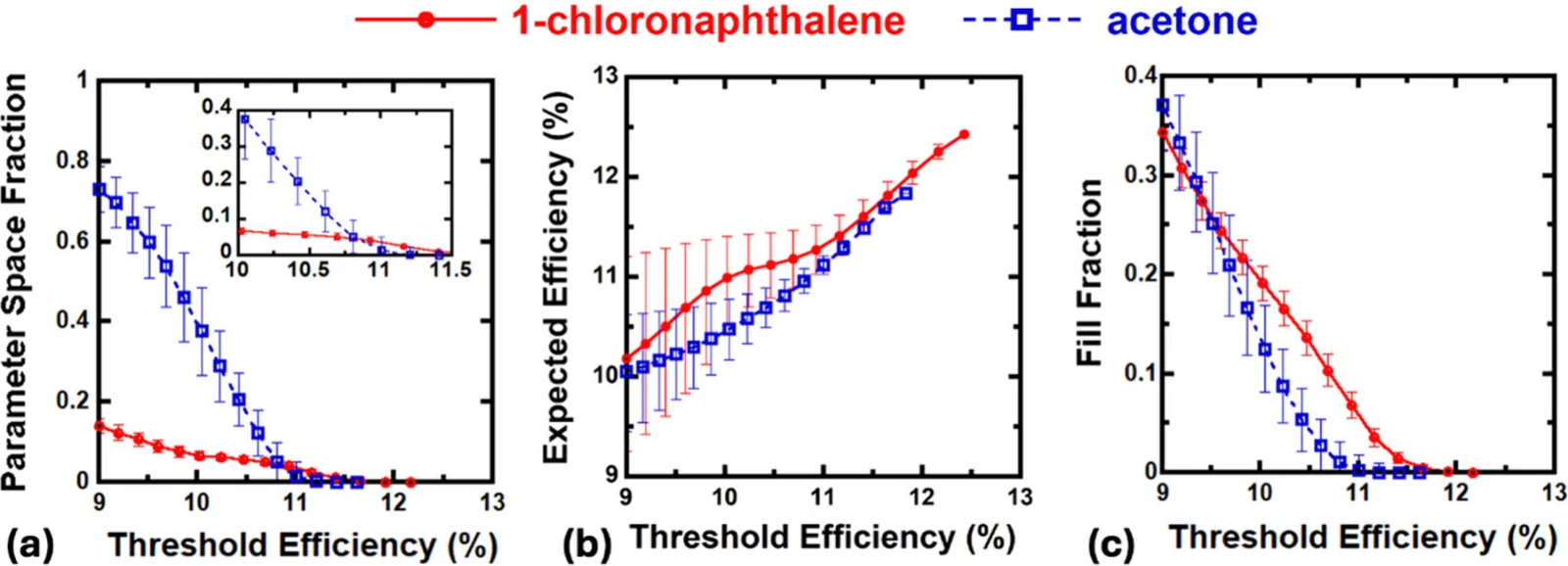
Persistence analysis of the acetone and 1-chloronaphthalene parameter space. (a) Parameter space fraction, (b) local mean expected efficiency, and (c) local optima fill fraction shown as functions of threshold efficiency. Error bars in (a) and (c) indicate surrogate prediction spread based on experimental variability from five experimental data sets. Error bars in (b) represent the 16th and 84th percentiles, corresponding to a ±1σ confidence interval for the primary component.

We also highlight the changes in local mean expected efficiency as a function of threshold efficiency. Local mean efficiency is taken within each component and represents the average efficiency of all possible photovoltaics that perform above a given threshold. The local mean expected efficiency for acetone increases steadily with threshold efficiency (Figure 3b). The 16th and 84th percentiles, corresponding to a ±1σ confidence interval, are shown as error bars in Figure 3b, serving as an indicator of predicted manufacturing consistency. The consistent curvature of the mean PCE indicates a smooth and well distributed local maximum without significant surface irregularities. Using acetone as an additive induces a more robust parameter space in PM6:Y6 OPVs, improving both the parameter space fraction and device consistency over 1-chloronaphthalene in exchange for lower overall performance. OPVs utilizing acetone additive are shown to have more robust and consistent processing, while 1-chloronaphthalene OPVs are more suitable when higher efficiencies are required.

We define a fill fraction as the ratio between the hypervolume of a contiguous high-performing region, and the hypervolume of the smallest prismatic region that fully encloses it. In three dimensions, this is analogous to the volume of a hill divided by the volume of the smallest prism that can contain it. Qualitatively, the fill fraction represents the curvature of the PCE landscape with flatter surfaces having fill fraction near unity. This measure of the processing robustness is thus more sensitive to the shape of the predicted value function near optimum, as opposed to the parameter space fraction, which depends more strongly on the total range of parameter space considered. Plotted in Figure 3c, the fill fraction of OPVs drops as threshold efficiency increases, indicating localized sharpening of the PCE landscape, as threshold efficiency increases. The crossover in between the acetone and 1-chloronapthalene curves (Figure 3c) occurs earlier than with the parameter space fraction (Figure 3a), where devices made with 1-chloronapthalene have a more gradual dependence on processing parameters as the threshold acceptable efficiency rises when compared to devices made with acetone for the same threshold OPV efficiency.

Devices fabricated with acetone exhibit a broad robust regime with ∼73% of the explored processing space yielding PCE > 9%. Partial dependence analysis further indicates that additive selection strongly influences model response, with partial dependence decreasing more quickly with the addition of 1-chloronaphthalene, suggesting a stronger drop in PCE with increasing amounts of this additive (Figures S3 and S4). One possible explanation is evaporation kinetics; the high volatility of acetone promotes rapid solvent removal during fabrication, whereas 1-chloronaphthalene can persist in the active layer. Residual 1-chloronaphthalene may perturb microstructure during subsequent steps, increasing variability through sensitivity to uncontrolled environmental factors. We hypothesize this would disrupt the balance between intermixed and pure phases in PM6:Y6, lowering performance.^46,47^ A complementary hypothesis is that acetone and 1-chloronaphthalene shape solvent quality differently during drying, which in turn influences polymer aggregation pathways.^48,49^ Together, our results highlight acetone as a robustness-inducing additive for PM6:Y6 OPVs with future mechanistic studies required to pinpoint specific drying and aggregation pathways create tolerant morphologies across diverse conditions.

Active learning approaches such as adaptive design of experiments (ADOE) have become the forefront techniques for implementation of sequential learning.^50,51^ The technique is built on the principles of Bayesian statistics, utilizing an acquisition function to recommend the next experimental conditions on demand.^52^ The controlled transition from exploration to exploitation ensures the model adequately surveys the parameter space. With further research and development, active learning techniques such as Bayesian driven ADOE are expected to replace static approaches when evaluating computationally expensive ’black box’ systems such as OPVs.^53,54^ Here, we used static DOE because examining robust parameter design required broad and thorough exploration of the parameter space without prior knowledge of the local optima. Knowledge and data gained in this work can inform future use of ADOE to explore the OPV parameter space.

High dimensionality and limited samples make OPV mapping challenging (the small-N, high-D modeling challenge). We evaluated randomized splits, k-fold, and leave-one-out cross-validation, but all assume similar train/test distributions; an assumption that becomes fragile at small N. A k-means, cluster-aware 80/20 split mitigated this issue and improved *R* ^2^ relative to random splits.^55^ Even so, the skewed distribution and wider PCE range observed with 1-chloronaphthalene (Figure 1) reduced surrogate fidelity. Additional targeted sampling in underrepresented regions of the 1-chloronaphthalene space would likely improve fit quality and enable sharper comparisons.

Use of ML and DOE to map the OPV parameter space can reveal potential areas of interest for fundamental study. Persistence analysis quantifies parameter space fraction, local mean efficiency, and fill fraction as functions of threshold efficiency, providing regional insight into rapid changes to the parameter space. The PCE landscape is generally expected to be both smooth and continuous with gradual gradients. Any rapid changes in PCE associated with specific processing conditions would therefore indicate regions of interest for subsequent study examining active layer properties. Raw collections of OPV optimization data can be difficult to interpret visually due to the large number of variables being tuned simultaneously. Machine learning enables the interpretation of these high dimensional data sets to create topographic maps like those seen in Figure 2. Connected components enable identification of contiguous local maxima. This permits experimenters to identify if a new set of conditions is truly independent from existing champion conditions. Collectively, machine learning and connected components provide intuitive insight into high dimensional design of experiments data sets.

Populating the space with a trained model requires choosing parameter range, resolution and scaling. Here, we used a 20-step linear grid per factor (20^5^) to balance inputs, with processing ranges chosen to reflect reasonable values for devices based on PM6:Y6 and other organic semiconductors. Initial parameter ranges (Table S1) can reflect current manufacturing capabilities or be narrowed to focus on a region of interest. The impacts of sampling method and parameter ranges were explored in Figures S5–S7 of the Supporting Information. For the cases we examined, minor changes to persistence curves were observed, but comparative conclusions remained consistent. This remained true provided most of the local maxima remained within the sampled parameter space. In a manufacturing context, the resolution within each variable can be scaled according to experimental precision. We explore this possibility by setting the number of points based on the experimental uncertainty of each processing variable, as shown in Table S2 and Figures S8 and S9 of the Supporting Information. For the data studied here, modification of the parameter range and sampling methods lead to minimal changes to qualitative persistence predictions; this highlights the suitability of evenly spacing the identified parameter space for initial analyses of processing robustness.

Overall, the mapping and metrics introduced here distinguish manufacturing-relevant robustness from peak PCE. Our work paired a design of experiments with machine-learning surrogates to map the OPV processing landscape and compare robustness using three complementary metrics: parameter-space fraction, persistence, and fill fraction. This framework turns high-dimensional DOE data into manufacturing-relevant measures and can be tuned to different priorities (e.g., yield vs peak PCE), providing practical guidance for choosing processing conditions and material combinations without additional experiments.

Applied to PM6:Y6, acetone as a solvent additive yields a broad, robust regime: ∼73% of sampled conditions exceed 9% PCE. In contrast, models trained on 1-chloronaphthalene data show lower fidelity, reflecting skewed coverage and wider performance spread; this underscores how initial data distribution shapes surrogate quality and suggests that additional targeted sampling would improve fit and enable more precise comparisons. This approach offers a general, data-efficient pathway to separate robustness from record PCE and accelerate translation toward scalable OPV fabrication.

## Supplementary Material



## Data Availability

Software used for this work can be found at https://github.com/ankush-kumar-mishra/Robustness_Analysis

## Notes

Additional details of experimental design, methods, and model performance comparisons (PDF)
