# Supplementary material for: Machine Learning Driven Analysis Yields Insight into the Processing Robustness of Organic Photovoltaics
Source: ACS Macro Lett. 2026 Jul 23;15(8):1117–22. doi: 10.1021/acsmacrolett.6c00284 (PMC13492376; doi:10.1021/acsmacrolett.6c00284)
Supplement: Supplementary file 1 [file mz6c00284_si_001.pdf]

Supporting Information for

Machine learning driven analysis yields insight into the processing robustness of organic photovoltaics

*Stephen Wong[1], Ankush Mishra[2], Baskar Ganapathysubramanian[2], and Enrique D. Gomez[1,3]**

[1]Department of Chemical Engineering, The Pennsylvania State University, University Park, PA 16802

[2]Department of Mechanical Engineering, Iowa State University, Ames, IA 50011, USA

[3]Materials Research Institute, The Pennsylvania State University, University Park, PA 16802

*email: edg12@psu.edu

Materials and Methods

Materials and Experimental Methods

Poly[(2,6-(4,8-bis(5-(2-ethylhexyl-3-fluoro)thiophen-2-yl)-benzo[1,2-b:4,5-b']dithiophene))-alt-(5,5-(1',3'-di-2-thienyl-5',7'-bis(2-ethylhexyl)benzo[1',2'-c:4',5'-c']dithiophene-4,8-dione)] (PM6), 2,2'-((2Z,2'Z)-((12,13-bis(2-ethylhexyl)-3,9-diundecyl-12,13-dihydro-[1,2,5]thiadiazolo[3,4-e]thieno[2'',3'':4',5']thieno[2',3':4,5]pyrrolo[3,2-g]thieno[2',3':4,5]thieno[3,2-b]indole-2,10-diyl)bis(methanylylidene))bis(5,6-difluoro-3-oxo-2,3-dihydro-1H-indene-2,1-diylidene))dimalononitrile (Y6), and N,N'-Bis(N,N-dimethylpropan-1-amine oxide)perylene-3,4,9,10-tetracarboxylic diimide (PDINO) were used as purchased from 1-Material. Clevios P 4083 poly(3,4-ethylenedioxythiophene) polystyrene sulfonate (PEDOT:PSS) was used as purchased from Heraeus Epurio.

All photovoltaics were fabricated in an ITO/PEDOT:PSS/PM6:Y6/PDINO/Al architecture. Indium doped tin oxide (ITO) glass substrates were sonicated in soapy water for 10 minutes and rinsed with deionized water. The substrates were then sonicated for 10 minutes in acetone then isopropanol and dried before cleaning under ultraviolet-ozone plasma treatment for 20 minutes. A PEDOT:PSS hole transport layer was filtered with 0.45 µm polyvinylidene fluoride and spin-coated onto the substrate at 2000 rpm for 30 seconds and annealed at 150°C for 15 minutes before cooling. Active layer solutions of PM6 and Y6 were prepared in chloroform with varying amounts of 1-chloronaphthalene or acetone solvent additive. Solutions were then allowed to stir for 3 hours at 45°C and 400 rpm. Active layer films were spin coated for 30 seconds and annealed for 10 minutes; both the spin speed and annealing temperature were determined via a static design of experiment strategy. An electron transport layer of PDINO were filtered through 0.2 µm polytetrafluoroethylene and deposited at 2500 rpm for 30 seconds using a 5 mg/mL solution in MeOH. The aluminum top contact was deposited by vacuum thermal evaporation; the first 10 nm deposited at 0.2 Å/s and the remaining 65 nm at 2.0 Å/s resulting in devices with an area of 0.162 cm^2.

Design of Experiments and Computational Approach

Five processing parameters (Table S1) were each discretized into five levels. We first generated 25 systematically varied recipes using a Taguchi orthogonal array (5 levels × 5 factors) in Minitab. To improve space-filling coverage, we added 32 points from a Sobol low-discrepancy sequence, linearly scaled to the parameter bounds. In total, this yielded 57 unique parameter combinations illustrated in Figure S1.

Table S1. Parameter space ranges

Parameters	**Ranges**
Donor wt. Ratio	0.5 – 1.5
Concentration (mg/mL)	8 – 22
Active Layer Spin Speed (rpm)	800 – 6000
Annealing Temperature (°C)	55 – 130
Solvent Additive (v/v %)	0.0 – 5.0

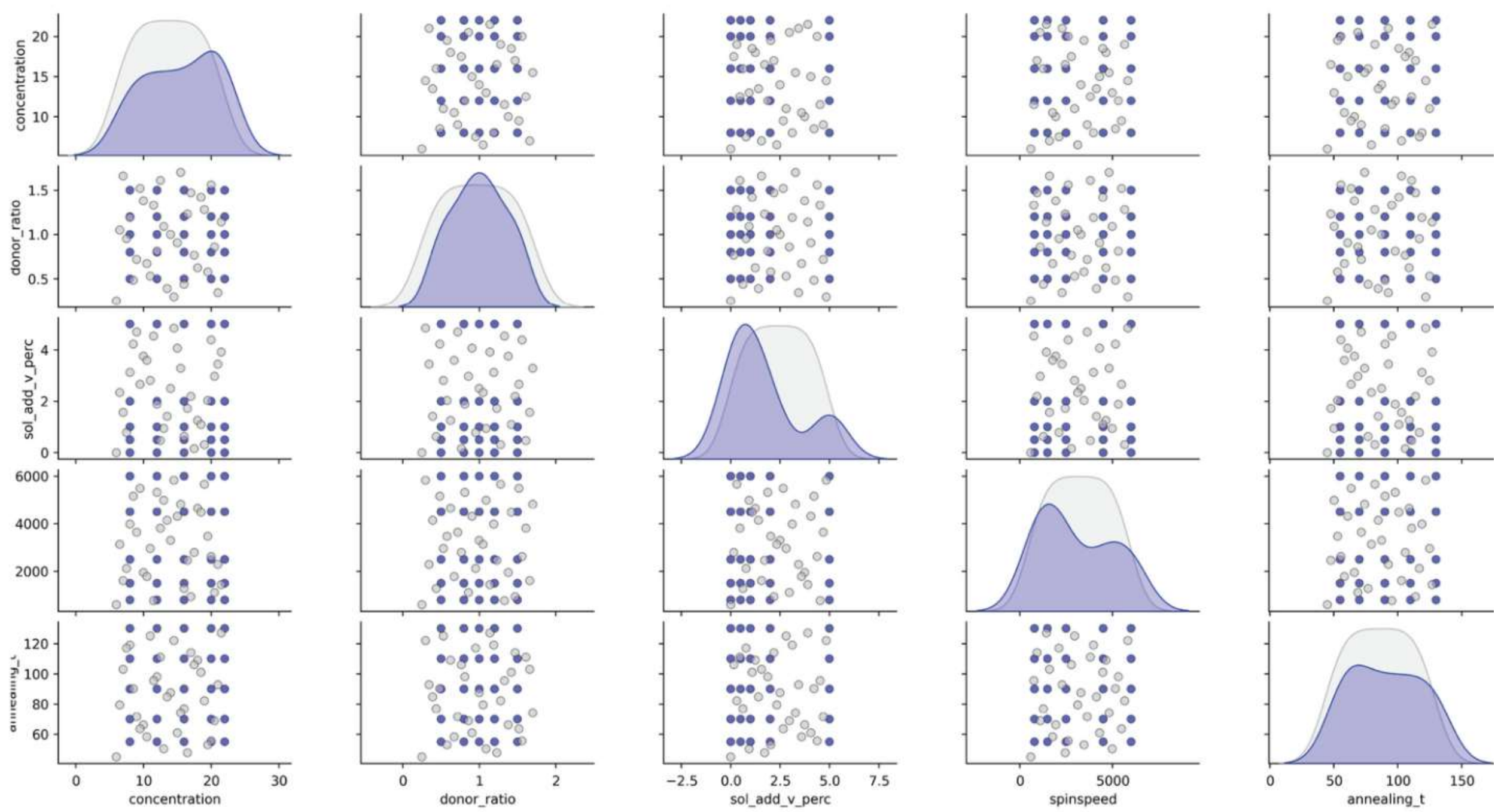

Figure S1: Combined Taguchi and Sobol Design of Experiments. Blue points were determined by Taguchi design while grey were determined by Sobol space-filling design. The rows and columns are concentration (mg/ml), donor wt. ratio, solvent additive v/v%, spin speed (rpm) and annealing temperature (°C) read top-to-bottom and left-to-right.

We evaluated several regressors, including random forest (RF), support vector regression (SVR), and gradient-boosting regression (GBR); the full list can be found in the next section. Each unique processing parameter combination (with replicates averaged) was treated as one data point. We created an 80/20 hold-out split with a k-means, cluster-aware strategy to reduce “lucky” splits and imbalance. The clusters were computed in the input (i.e., processing parameter) space, the

number of clusters was chosen by the elbow method (k = 8), and the train/test split preserved cluster proportions. Given that each condition had 12 to 18 datapoints, we estimate the prediction uncertainty by training models on five different sets of datapoints, where each datapoint is chosen randomly from total available data at each combination of processing conditions. The resulting standard deviations of these predictions was reported as error bars in Figures 3a and 3c.

Performance on the held-out test set was compared across models using R^2 and mean-squared error (MSE) (Figure S2), after which RF was selected for downstream analysis. Several machine learning algorithms were evaluated during model training and diagnostics. These included linear regression (LR), k-nearest neighbors (kNN), support vector regression (SVR), random forest (RF), adaboost, gradient boosting regressors (GBR), bagging regressors, extra trees regressors, and histogram-based gradient boosting regressors. Implementing hierarchal data handling during train/test splits and appropriate trial weighting proved to be the main limitation. GBR, RF, and SVR consistently performed well during initial evaluation, and were the primary focus for model optimization. RF was chosen to represent PM6:Y6 OPV performance after achieving the highest R^2 in both acetone and 1-chloronaphthalene containing devices. Figure S2 show R^2 and mean squared error (MSE) for each optimized model. Model quality was assessed by determining the accuracy of our trained models in predicting performance from our 80/20 held-out data split.

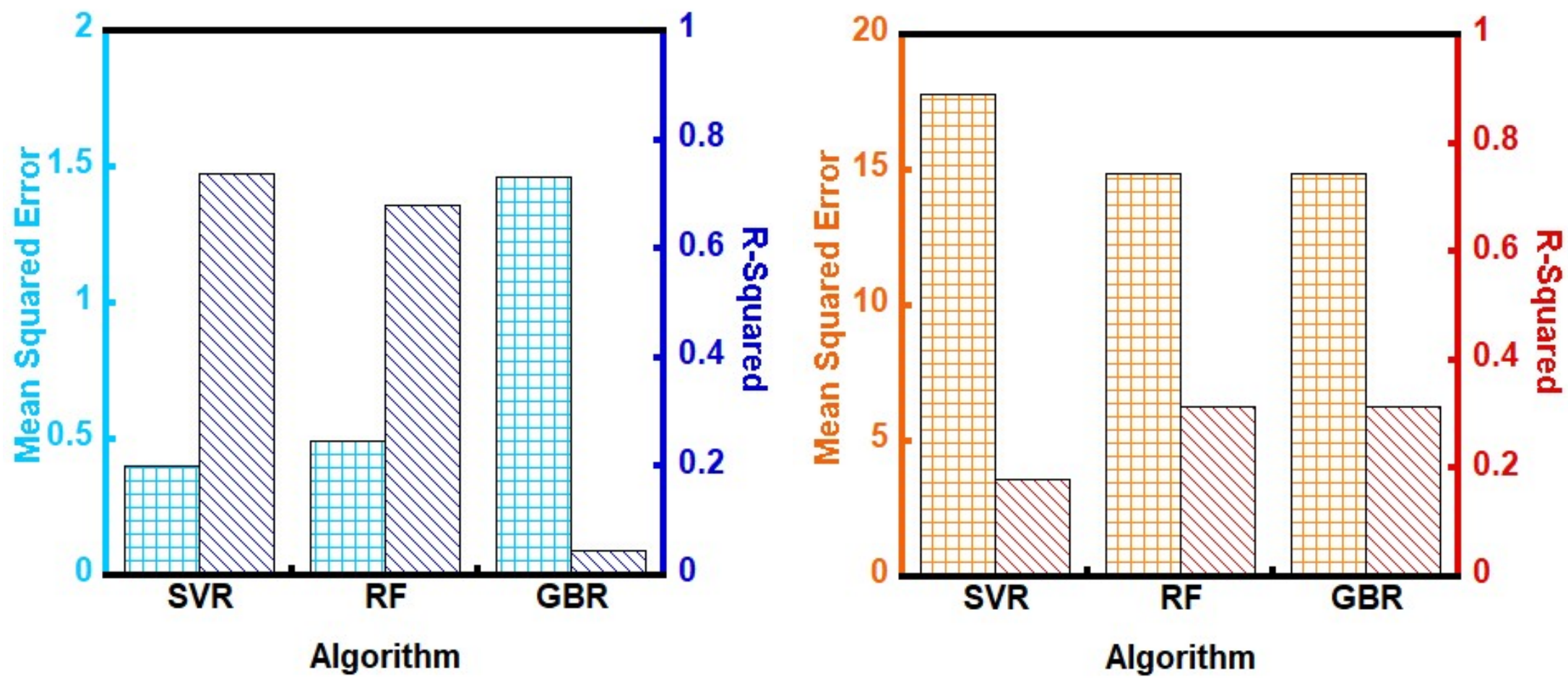

Figure S2: Model Performance for solvent additive PM6:Y6 OPV with k-means determined splitting. (Left) acetone additive (Right) 1-chloronaphthalene additive. Additional data collection is required to improve 1-chloronaphthalene model quality.

After selecting the ML model, we uniformly sampled the processing space on a 20-level grid per factor (i.e., a 20^5 mesh) and used the surrogate to predict PCE at each grid point. This gives us a densely sampled multidimensional PCE surface as a function of the 5 variables. Applying a PCE threshold of 9.0% on this surface produced a binary mask of "acceptable" processing conditions. We then identified contiguous high-PCE domains ("connected components") with a standard depth-first search (DFS) on the grid. For each component, we computed: (i) parameter-space fraction, the fraction of the global grid contained in that component; (ii) mean PCE, the average predicted PCE over points in the component; and (iii) fill fraction, which scores how uniformly high the region is by linearly scaling the PCE of each point between the component maximum and the threshold, averaging over the component. Fill fractions near 1 indicate broad, flat high-PCE plateaus; values near 0 indicate narrow peaks. Increasing the PCE threshold stepwise yields persistence curves that track how these regions contract as stricter performance is demanded.

All analyses were performed in Python 3 using pandas, scikit-learn, and SciPy; visualizations used Matplotlib (and seaborn for styling). All analysis code and Jupyter notebooks used in this work will be publicly available upon publication.

Partial Dependence Parameter Analysis

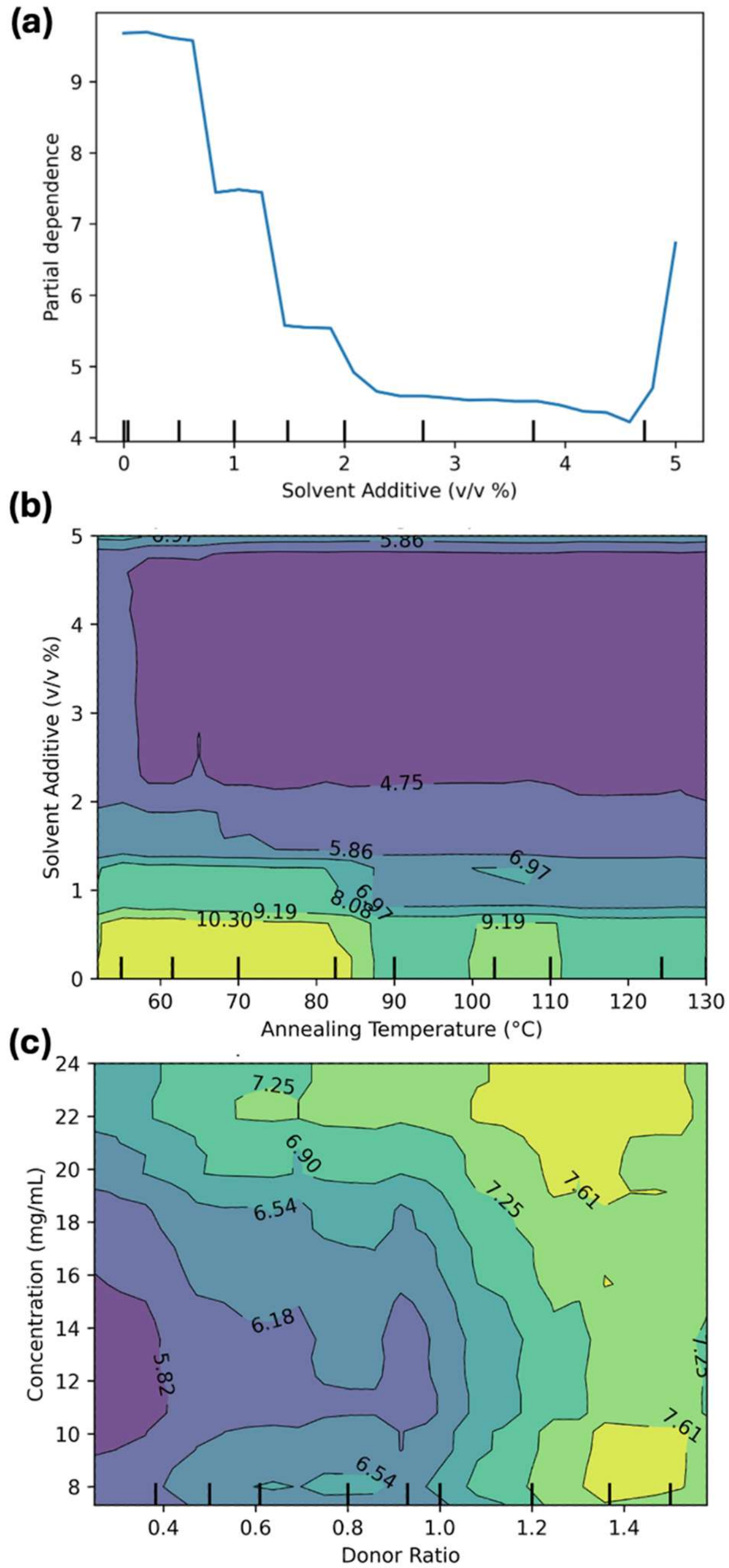

Figure S3: Partial dependence analysis of random forest fit to data from PM6:Y6 OPVs made with 1-chloronaphthalene as an additive. (a) Partial dependence on solvent additive v/v%, (b) solvent additive v/v% and annealing temperature (°C), and (c) concentration and donor ratio. Bold vertical lines denote location of experimental data (Rug plot).

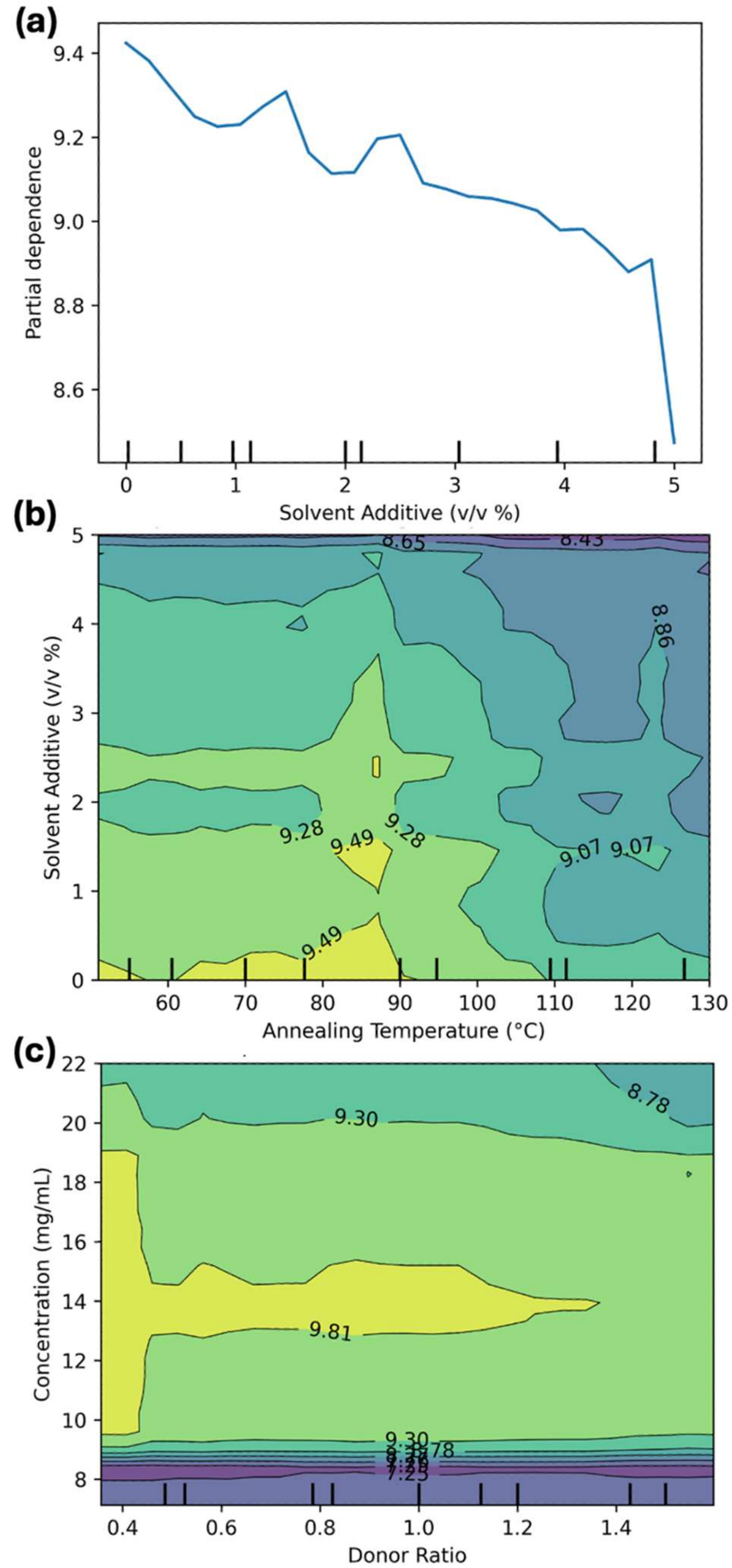

Figure S4: Partial dependence analysis of random forest fit to data from PM6:Y6 OPVs made with acetone as an additive. (a) Partial dependence on solvent additive v/v%, (b) solvent additive v/v% and annealing temperature (°C), and (c) concentration and donor ratio. Bold vertical lines denote location of experimental data (Rug plot)

Impact of User-Defined Parameters on Persistence Analysis

A 20-step per factor linear grid (20^5) was used as a baseline to equally represent all inputs and values in our baseline analysis. Steps size be linear, logarithmic, or exponential to emphasize regions of interest; for example, additive v/v% often exerts its strongest influence at 0–1%, where log-spaced steps provide additional detail.

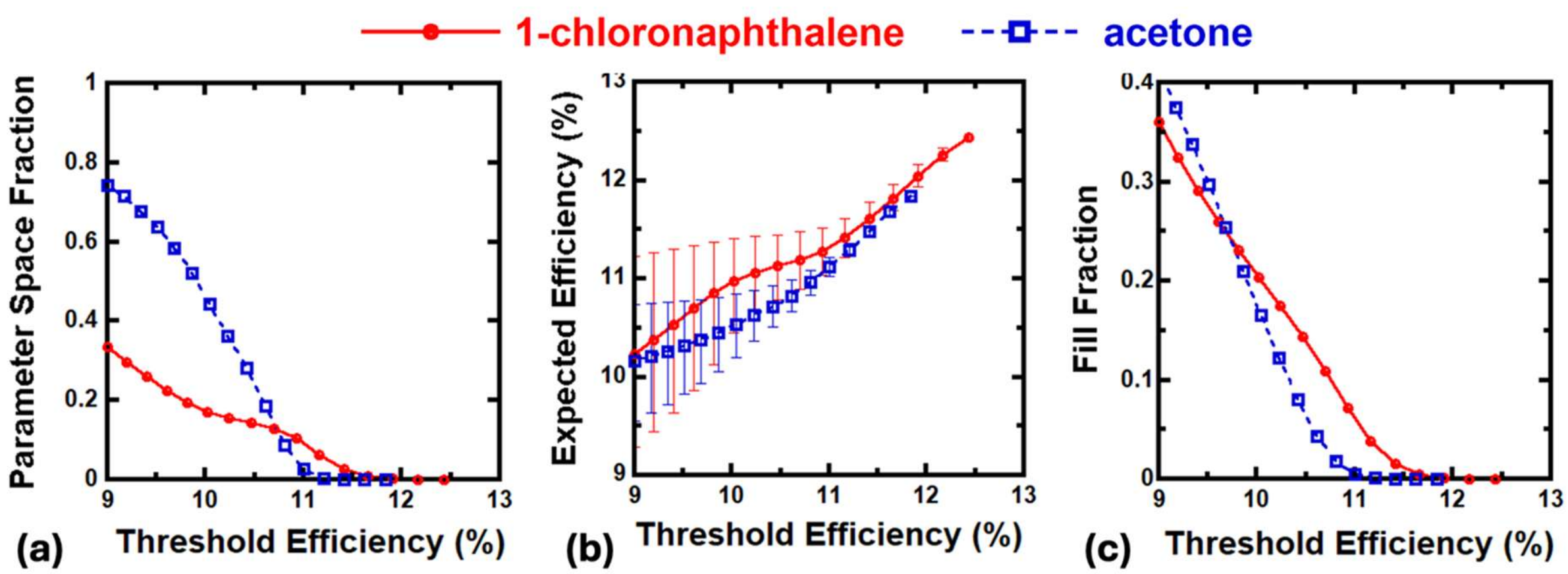

Figure S5: Persistence analysis of the acetone and 1-chloronaphthalene with logarithmic additive sampling. (a) parameter space fraction, (b) local mean expected efficiency, (c) local optima fill fraction shown as functions of threshold efficiency. Error bars represent the ±1σ confidence interval for the primary component.

To examine the impacts of logarithmic sampling we maintained the 20-step resolution for our analysis, with logarithmic sampling of the solvent additive parameter and depicted above (Figure S5). The conclusions drawn by comparing the 1-chloronaphthalene and acetone persistence curves remain consistent with those of Figure 3. The most significant difference can be found in Figure S5a where the 1-chloronaphthalene parameter space fraction increases substantially at low efficiencies. This is a direct result of the additional sampling provided at low additive v/v %, and possibly due to the strong impact of trace 1-chloronaphthalene on PCE (Figure S3a).

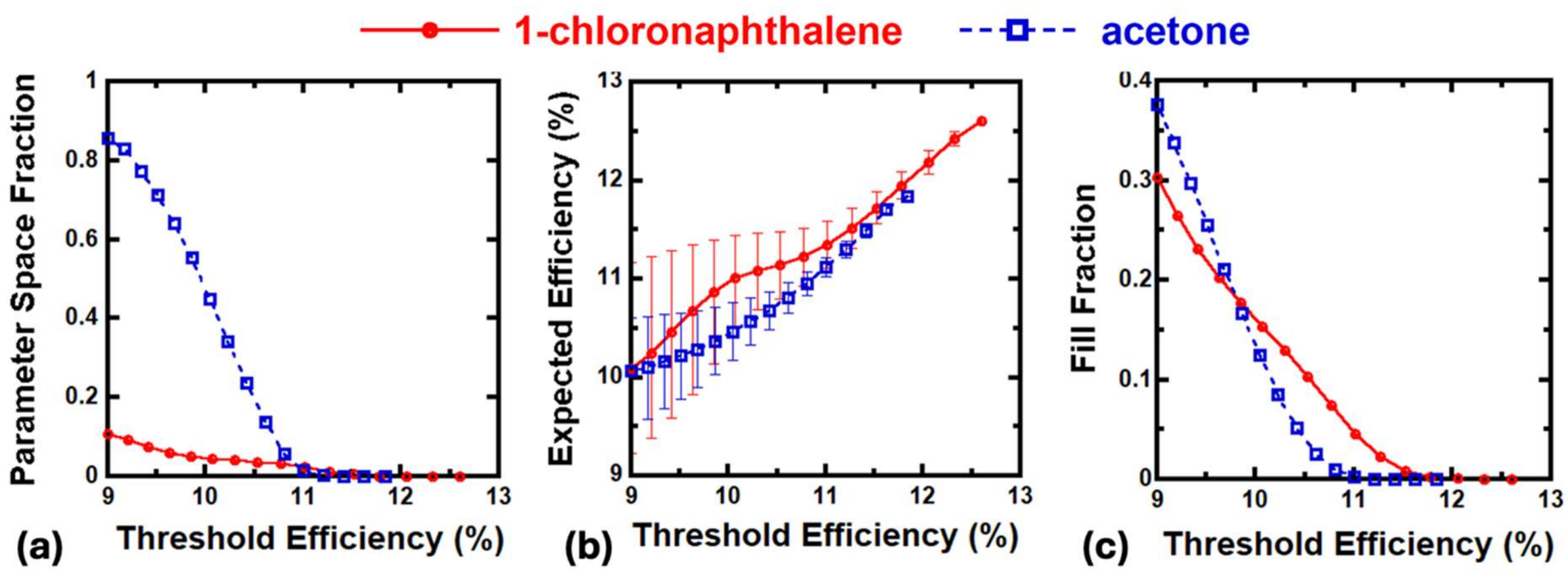

Figure S6: Persistence analysis of the acetone and 1-chloronaphthalene with 10% reduced parameter range. (a) parameter space fraction, (b) local mean expected efficiency, (c) local optima fill fraction shown as functions of threshold efficiency. Error bars represent the ±1σ confidence interval for the primary component.

Choice of parameter range can influence both experimental requirements and the exact details of resulting persistence analyses. Figure S7 illustrates a version of our framework completed using the same training data and machine learning models, where the parameter space mapped has been reduced by 10% on each axis. The sampling remains a 20-step linear grid over all parameters, and the center of the parameter space center has been maintained. With a zoom of only 10% applied, the persistence curves (Figure S6a-c) remain largely consistent with prior analysis. An increase in absolute parameter space fraction is expected due to a component of similar size taking up a larger portion of a smaller sampled parameter space. While this effect is observed for acetone additive (Figure S6a), it is less pronounced for 1-chloronaphthalene due to the position of the local maxima position near the edge of the parameter space. Narrowing of the parameter ranges thus cut off some of the connected component we desired to measure, minorly impacting the 1-chloronaphthalene parameter space fraction, and highlighting the need for parameter ranges to be sufficiently wide to capture local optima.

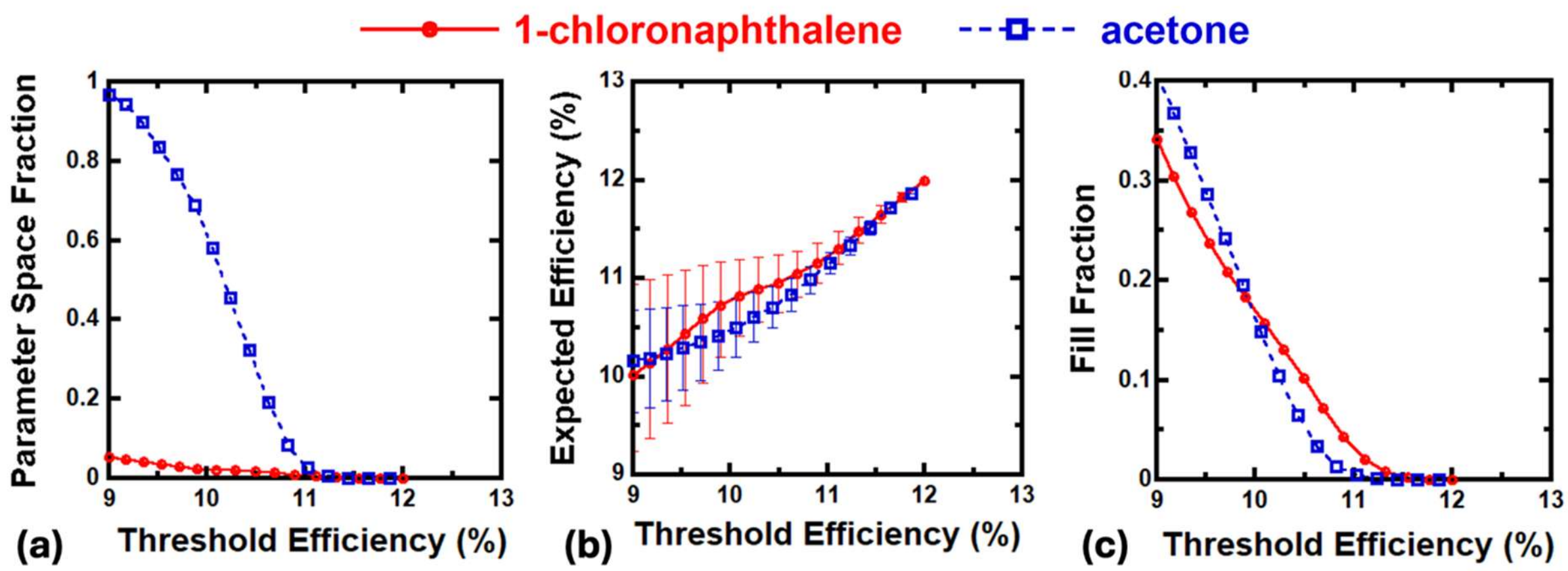

Figure S7: Persistence analysis of the acetone and 1-chloronaphthalene with 25% reduced parameter range. (a) parameter space fraction, (b) local mean expected efficiency, (c) local optima fill fraction shown as functions of threshold efficiency. Error bars represent the ±1σ confidence interval for the primary component.

Modification of parameter ranges impacts analysis more substantially when the zoom was increased to 25%, due to reducing the sampled parameter space even further and incomplete evaluation of local maxima excluded from the reduced ranges. The most significant changes occur when parameter ranges are narrowed sufficiently to cause the partial or complete elimination of a connected component. This is visible in the distortion of the 1-chloronaphthalene persistence curve (Figure S7a), where parameter space fraction has decreased despite reductions in overall parameter space sampled. In these cases, adjustments to parameter ranges and sampling did not influence qualitative conclusions drawn from comparisons between consistently configured parameter spaces. This remains consistent as long as most of the connected components remain within the ranges evaluated.

Scaling of Parameter Space According to Experimental Error

Table S2. Parameter space scaling to experimental precision

Parameters	**Number of points (levels)**	
	**Based on experimental precision**	**Alternative rescaling**
Donor wt. Ratio	9	30
Concentration (mg/mL)	13	49
Active Layer Spin Speed (rpm)	43	10
Annealing Temperature (°C)	9	10
Solvent Additive (v/v %)	6	19

Scaling of the parameter space according to experimental error would allow this framework to better assess the accessibility of promising processing conditions. The original parameter space was done according to a uniform 20-point linear sampling with 20^5 overall points surveyed according to our trained models. To evaluate the feasibility of certain optimal processing conditions for manufacture, we explored scaling of the parameter space based on equipment error as collected by manufacturers such as Ossila and Eppendorf. Propagation of error was used to determine the error of derived parameters throughout their ranges, and the resolutions of each axis were generated and scaled procedurally based on the precision of a given parameter and available computational resources. Solution batch sizes were assumed to be 1 mL, and alternate parameter assigned mean values where necessary. The scaling factor (k) functioned as a coefficient applied to the propagation of error at a given point, with 1=k representing approximately ±1σ error and 2=k representing ±2σ and increasing as necessary to lessen computational burden.

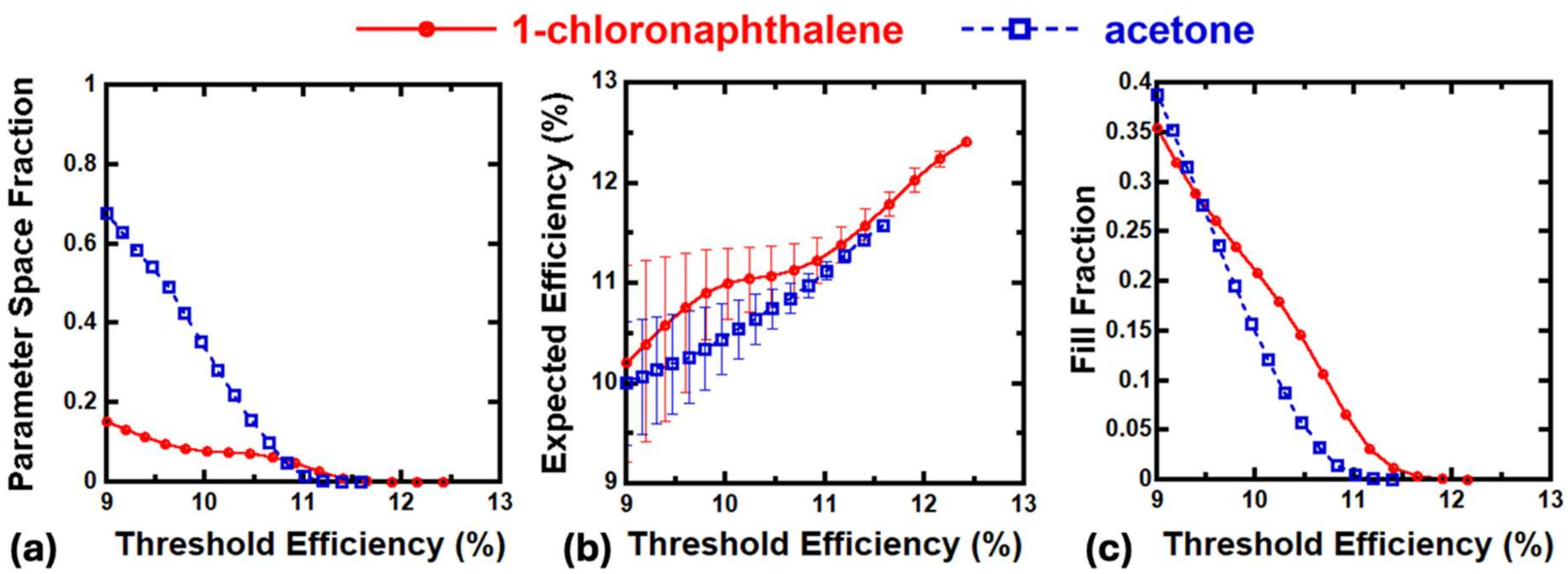

Figure S8: Persistence analysis of the acetone and 1-chloronaphthalene accessible parameter space. (a) parameter space fraction, (b) local mean expected efficiency, (c) local optima fill fraction shown as functions of threshold efficiency. Error bars represent the ±1σ confidence interval for the primary component.

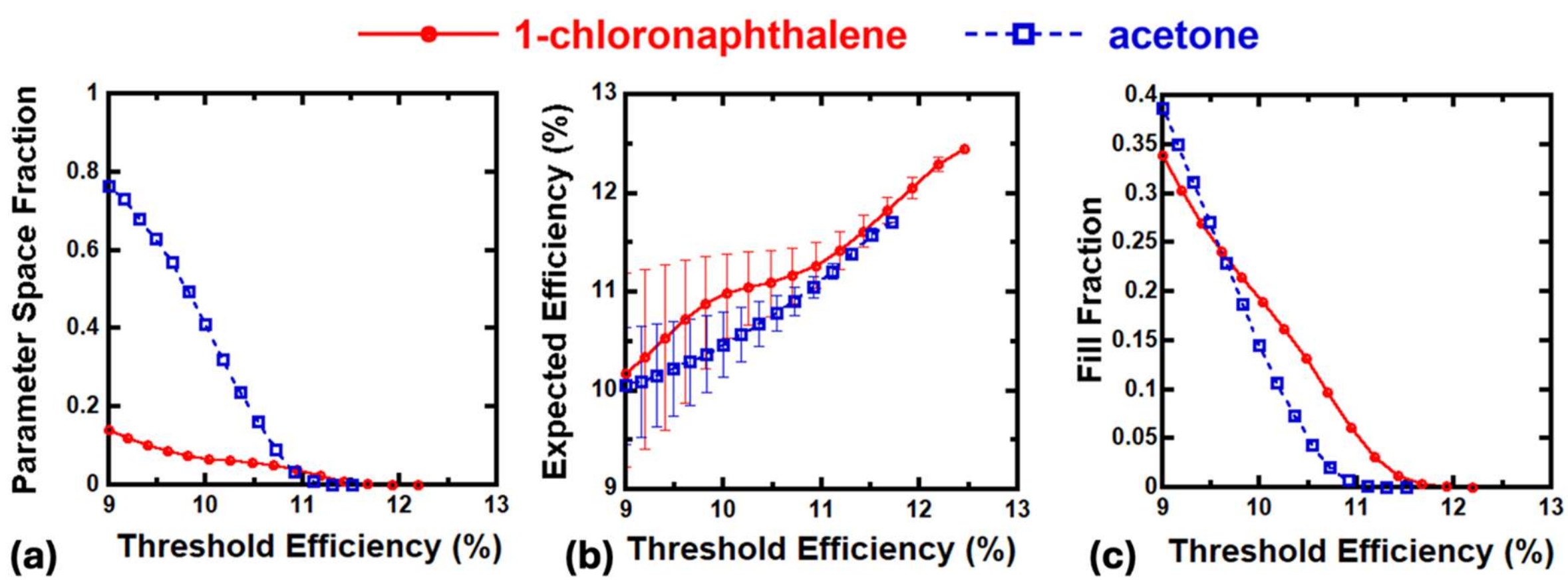

Figure S9: Persistence analysis using 10 step resolution cap on spin-speed and annealing temperature parameters. (a) parameter space fraction, (b) local mean expected efficiency, (c) local optima fill fraction shown as functions of threshold efficiency. Error bars represent the ±1σ confidence interval for the primary component.

Varying degrees of available experimental precision could lead to overrepresentation of specific parameters. We rescale parameters using intuition developed from the literature on organic solar cells, where donor ratio, concentration, and additive content are known to play a substantial role. We then set spin speed and annealing temperature to 10 values (Table S2). The robustness predictions drawn from scaled parameter spaces (Figures S5 and S6) resemble strongly the

predictions made using uniform scaling (Figure 3). Experimental error-based scaling of the parameter space proved to have minor impacts on this comparative analysis of PM6:Y6 OPVs with acetone and 1-chloronaphthalene additives. We expect that this nonlinear approach to resolution scaling could have more pronounced repercussion on analyses within systems containing multiple local maxima of similar size.
